# Clinical characteristics and associated factors of cholestasis in infant’s ≤3 months with cytomegalovirus infection

**DOI:** 10.3389/fmicb.2026.1860504

**Published:** 2026-06-25

**Authors:** Wenmei Li, Kun Wang, Haifeng Geng, Xinjing Lv, Caimei Li, Qinghui Chen, Fangfang Cheng, Weifang Zhou, Jianmei Tian, Huawei Wang, Lin Che

**Affiliations:** 1 Department of Neonatology, Children’s Hospital of Soochow University, Suzhou, China; 2 Department of Infectious Diseases, Children’s Hospital of Soochow University, Suzhou, China

**Keywords:** cholestasis, cytomegalovirus, immune profile, infant, liver function

## Abstract

**Objective:**

The clinical characteristics and contributing factors of cholestasis in infants with cytomegalovirus (CMV) infection remain incompletely understood. This study aimed to investigate the clinical features and associated factors of cholestasis in infants with CMV infection.

**Methods:**

This retrospective case–control study enrolled infants aged ≤3 months with confirmed CMV infection. Infants with cholestasis were classified as cases, and age-matched infants without cholestasis were randomly selected as controls in a 1:1 ratio. Clinical and laboratory characteristics were compared between groups. Factors independently associated with cholestasis were identified using multivariable logistic regression analysis, and a predictive nomogram was constructed accordingly. Furthermore, an exploratory phenotype axis framework was applied to delineate the multidimensional clinical characteristics of CMV-infected infants.

**Results:**

A total of 64 infants with CMV infection and cholestasis were included. Compared with controls, infants with cholestasis had a later onset and longer duration of jaundice, a lower rate of passing hearing screening, and longer hospital stays (all *p* < 0.05). Multivariable logistic regression revealed that delayed onset of jaundice was independently associated with cholestasis (aOR 1.247, 95% CI 1.101–1.412), whereas breastfeeding (aOR 0.408, 95% CI 0.159–0.840), higher total protein levels (aOR 0.882, 95% CI 0.797–0.976), and higher CD4^+^/CD8^+^ ratios (aOR 0.589, 95% CI 0.365–0.951) were inversely associated with the occurrence of cholestasis. Exploratory analysis indicated that infants with cholestasis exhibited a higher overall burden across multidimensional clinical phenotypes.

**Conclusion:**

In infants with CMV infection, delayed onset of jaundice was independently associated with cholestasis, whereas higher total protein levels, higher CD4^+^/CD8^+^ ratios, and breastfeeding were associated with a lower risk.

## 1 Introduction

Cytomegalovirus (CMV) is a common and clinically significant pathogen in infancy, capable of affecting multiple organ systems. The clinical manifestations of CMV infection are highly variable and heterogeneous, with considerable differences in severity (Tzialla et al., 2025). Previous studies have shown that most infected infants lack specific symptoms, whereas symptomatic cases present a highly heterogeneous clinical spectrum, substantially complicating disease recognition and risk stratification (Tzialla et al., 2025; Salomè et al., 2025). The clinical presentation of CMV infection in infants is diverse, involving multiple organ systems, including hearing impairment, ocular abnormalities, anemia, elevated liver transaminases, jaundice, and hepatosplenomegaly; a subset of affected infants may further develop cholestasis (Tzialla et al., 2025; Sun et al., 2025; Kenneson and Cannon, 2007). Among these, jaundice and cholestasis are particularly common in young infants, though their severity and clinical course vary considerably (Hasosah et al., 2012; Min et al., 2017). Cholestasis, as an important clinical syndrome in infancy, involves multiple mechanisms including infection, biliary tract abnormalities, and immune-mediated inflammation (Ranucci et al., 2022). Notably, although CMV infection can be detected in some infants with cholestasis, viral positivity does not correspond directly to disease severity, suggesting that host factors or other regulatory mechanisms may play a key role in disease progression (Salomè et al., 2025; Bryant et al., 2002; Zhao et al., 2021).

Therefore, identifying infants at higher risk of developing cholestasis in the context of CMV infection has greater clinical significance than relying solely on virological testing. However, existing studies have primarily focused on pathogen detection, isolated liver function markers, or specific complications, and systematic assessment of cholestasis as a clinical phenotype remains limited. In particular, integrated studies evaluating the risk of cholestasis in young infants, combining clinical manifestations, biochemical indicators, and immune status, are still lacking.

Given the limited understanding of CMV-associated cholestasis in early infancy, this study aimed to comprehensively characterize the clinical features of CMV-infected infants aged ≤3 months with and without cholestasis and to identify factors independently associated with its occurrence. Furthermore, multidimensional clinical parameters were integrated to assess hepatobiliary dysfunction, metabolic disturbances, immune alterations, and extrahepatic manifestations. By providing a more comprehensive characterization of CMV-associated cholestasis, this study may contribute to improved risk assessment and support earlier recognition and management of affected infants.

## 2 Methods

### 2.1 Study design and participants

This single-center retrospective case–control study, conducted at the Children’s Hospital of Soochow University, a large tertiary teaching hospital and the regional referral center for pediatric care in southern Jiangsu Province with approximately 1,500 inpatient beds and more than 2.5 million outpatient visits annually, included infants aged ≤3 months who were admitted to this institution between January 1, 2020, and December 31, 2024, with a diagnosis of CMV infection. Infants with cholestasis were assigned to the case group, and age-matched infants without cholestasis were randomly selected as controls in a 1:1 ratio. The median age at admission of the enrolled infants was 28.50 (IQR 20.00–35.50) days. Clinical data from both groups were compared to identify factors associated with the development of cholestasis.

The study protocol was approved by the Ethics Committee of the Children’s Hospital of Soochow University (Approval No. 2024CS098) and was conducted in accordance with the Declaration of Helsinki (1964) and its subsequent amendments, or other comparable ethical standards. Written informed consent was obtained from the legal guardians of all participants for the use and disclosure of clinical data.

### 2.2 Exclusion criteria

Infants were excluded if they had: (1) biliary atresia, choledochal cyst, or other structural biliary anomalies; (2) genetic or metabolic liver diseases; (3) other infectious diseases or severe systemic infections known to cause cholestasis; (4) primary immunodeficiency, severe congenital malformations, or chromosomal abnormalities; (5) prior hepatobiliary surgical interventions; or (6) missing critical clinical or laboratory data precluding outcome assessment. All exclusions were determined based on medical records during case selection.

### 2.3 Diagnostic criteria

CMV infection was defined as a positive CMV-DNA result in blood, urine, or other body fluids. Histopathological evidence of CMV inclusion bodies was also considered diagnostic (Leruez-Ville et al., 2024; Hao, 2018). Cholestasis was defined according to pediatric guidelines (Ranucci et al., 2022; Feldman and Sokol, 2013). Congenital cytomegalovirus infection (cCMV) was defined as the presence of CMV DNA detected in urine, saliva, blood, or cerebrospinal fluid samples, or a positive viral culture, obtained within the first 21 days of life. Breastfeeding was defined as exclusive or partial breast milk feeding from birth until the confirmation of CMV infection. Jaundice onset was defined as the postnatal age (in days) at which clinically visible jaundice was first documented.

### 2.4 Data collection

Clinical data were independently extracted from the hospital electronic medical record system by two investigators and cross-verified; discrepancies were resolved by a third investigator. Short-term follow-up data after discharge were supplemented via outpatient records. Collected variables included: (1) baseline and perinatal characteristics (sex, age at admission, age at onset, birth weight, admission weight, gestational age, delivery mode, Apgar scores, maternal infection during pregnancy, and perinatal complications); (2) laboratory parameters, including initial complete blood count, biochemical tests, liver function, and lymphocyte subsets, used as baseline variables; peak values during hospitalization were recorded to reflect disease severity but were not included in predictive modeling; (3) clinical manifestations and outcomes, including length of hospital stay, hepatosplenomegaly, onset and resolution of jaundice, comorbidities, and complications; (4) imaging data, including liver and biliary ultrasound and other relevant imaging examinations during hospitalization. (5) Outpatient follow-up visits were typically scheduled at approximately 2 weeks, 1 and 3 months after discharge. The variables retrieved from the follow-up records included the resolution or persistence of jaundice and its timing, weight gain, and the infant’s overall growth and development as documented by the attending physician.

### 2.5 Phenotype axis analysis

To systematically evaluate the multidimensional clinical characteristics of infants with CMV infection and cholestasis, an exploratory phenotype axis framework was developed based on prior literature (Feldman and Sokol, 2013; Ekman et al., 2024; Bilavsky et al., 2015; Karandikar et al., 2023; Payne et al., 2026) and the biological relevance of each variable. Variables were grouped into four axes: (1) Hepatobiliary axis, including jaundice onset and duration, direct bilirubin, alanine aminotransferase (ALT), aspartate aminotransferase (AST), gamma-glutamyl transferase (GGT), and abnormal liver ultrasound, reflecting cholestasis severity and hepatocellular injury; (2) Metabolic axis, including total protein, albumin, and cholinesterase, assessing hepatic synthetic function and metabolic imbalance; (3) Immune axis, including absolute lymphocyte count, CD3^+^CD8^+^ T cells, CD3^−^CD(16^+^56^+^) natural killer (NK) cells, and CD4^+^/CD8^+^ ratio, reflecting cellular immune status in CMV infection; (4) Extrahepatic involvement axis (Extrahepatic axis), including splenomegaly, intracranial hemorrhage, abnormal hearing screening, and ocular findings, evaluating multisystem involvement beyond the liver. This analysis was exploratory and aimed to describe multidimensional clinical phenotypic distributions.

### 2.6 Statistical analysis

Statistical analyses were performed using SPSS version 27.0. Continuous variables were assessed for normality; normally distributed variables are presented as mean ± standard deviation (SD) and compared using independent-sample t-tests, while non-normally distributed variables are presented as median (interquartile range, IQR) and compared using the Mann–Whitney U test. Categorical variables are expressed as counts and percentages and compared using the *χ*^2^ test or Fisher’s exact test, as appropriate.

Cholestasis was treated as the dependent variable. Variables with *p* < 0.05 in univariable analysis and deemed clinically relevant were included in a multivariable logistic regression model. Gestational age was included as a forced covariate. Variables directly related to the diagnosis of cholestasis were excluded to avoid circular reasoning. Based on the multivariable logistic regression model, a nomogram was constructed, and its discriminative ability was evaluated using the C-index, with calibration assessed via the Hosmer–Lemeshow test.

For the phenotype axis analysis, continuous variables were standardized (Z-scores), and inversely oriented variables were reversed for consistency. Axis scores were calculated as the mean of standardized variables within each axis, and overall phenotype burden was calculated as the average of the four axes. Group comparisons were performed using the Mann–Whitney U test. All tests were two-sided, and *p* < 0.05 was considered statistically significant.

## 3 Results

### 3.1 Baseline characteristics of infants with CMV infection

During the study period, a total of 392 infants met the inclusion and exclusion criteria for CMV infection. Among them, 64 infants were diagnosed with cholestasis, and 64 age-matched CMV-infected infants who met the inclusion criteria were randomly selected as controls at a 1:1 ratio. Baseline and perinatal characteristics of the two groups are summarized in Table 1. No significant differences were observed between the cholestasis and non-cholestasis groups with respect to gestational age, birth weight, sex, mode of delivery, birth asphyxia, age and weight at admission, patent ductus arteriosus, atrial septal defect, or ventricular septal defect (all *p* > 0.05). Maternal perinatal factors, including maternal age, premature rupture of membranes, meconium-stained amniotic fluid, gestational diabetes mellitus, hypertensive disorders of pregnancy, preeclampsia, perinatal infection, maternal hypothyroidism, antenatal steroid use, placental abruption, and intrahepatic cholestasis of pregnancy, also did not differ significantly between the two groups (all *p* > 0.05). Notably, the proportion of infants receiving breastfeeding was significantly lower in the cholestasis group compared with the non-cholestasis group (*p* < 0.05).

**Table 1:** Perinatal and baseline characteristics of infants with CMV infection.

Variable	Cholestasis group (*n* = 64)	Non-cholestasis group (*n* = 64)	*p*
Demographic characteristics			
Gestational age (weeks)	37.00 (35.00, 38.00)	36.00 (35.00, 38.00)	0.295
Birth weight (kg)	2.64 ± 0.39	2.56 ± 0.39	0.275
Male, *n* (%)	44 (68.8)	36 (56.2)	0.144
Cesarean delivery, *n* (%)	33 (51.6)	27 (42.2)	0.288
Birth asphyxia, *n* (%)	3 (4.7)	2 (3.1)	1.000
Age at admission (days)	26.50 (19.00, 35.00)	30.00 (21.00, 36.00)	0.436
Weight at admission (kg)	2.86 ± 0.45	2.80 ± 0.50	0.481
Breastfeeding, *n* (%)	12 (18.8)	27 (42.2)	0.004
Patent ductus arteriosus, *n* (%)	1(1.6)	3 (4.7)	0.619
Atrial septal defect, *n* (%)	19 (29.7)	18 (28.1)	0.845
Ventricular septal defect, *n* (%)	2 (3.1)	1 (1.6)	1.000
Perinatal characteristics			
Maternal age (years)	28.00 (26.00, 31.00)	27.50 (25.75, 30.00)	0.106
PROM (>18 h), *n* (%)	5 (7.8)	3 (4.7)	0.718
MSAF, *n* (%)	6 (9.4)	8 (12.5)	0.571
GDM, *n* (%)	11 (17.2)	11 (17.2)	1.000
HDP, *n* (%)	12 (18.8)	17 (26.6)	0.291
Preeclampsia, *n* (%)	11 (17.2)	9 (14.1)	0.626
Perinatal infection, *n* (%)	3 (4.7)	0 (0.0)	0.244
Maternal hypothyroidism, *n* (%)	0 (0.0)	1 (1.6)	1.000
Antenatal corticosteroid use, *n* (%)	2 (3.1)	0 (0.0)	0.496
Placental abruption, *n* (%)	3 (4.7)	4 (6.2)	1.000
ICP, *n* (%)	7 (10.9)	3 (4.7)	0.188
Congenital CMV	14 (21.9)	10 (15.6)	0.365

CMV, cytomegalovirus; PROM, premature rupture of membranes; MSAF, meconium-stained amniotic fluid; GDM, gestational diabetes mellitus; HDP, hypertensive disorders of pregnancy; ICP, intrahepatic cholestasis of pregnancy. Non-normally distributed continuous variables are expressed as median (interquartile range, IQR) and were compared using the Mann–Whitney U test; normally distributed continuous variables are expressed as mean ± SD and compared using the independent-sample *t*-test; categorical variables are expressed as *n* (%) and compared using the *χ*^2^ test or Fisher’s exact test, as appropriate.

### 3.2 Laboratory findings of infants with CMV infection

Laboratory data and lymphocyte subset analysis for both groups are presented in Table 2. Regarding hepatobiliary injury markers, infants in the cholestasis group had significantly higher levels of total bilirubin (TBil), direct bilirubin (DBil), indirect bilirubin (IBil), gamma-glutamyl transferase (GGT), alanine aminotransferase (ALT), aspartate aminotransferase (AST), and alkaline phosphatase (ALP) compared with the non-cholestasis group (all *p* < 0.05). No significant differences were observed in routine hematologic parameters, including white blood cell count, hemoglobin, platelet count, absolute neutrophil count, eosinophil percentage, and monocyte count (all *p* > 0.05). However, the cholestasis group had a significantly higher absolute lymphocyte count (*p* < 0.05). For other biochemical markers, total protein and cholinesterase levels were significantly lower in the cholestasis group compared with controls (*p* < 0.05), whereas albumin, creatine kinase (CK), lipase, and lactate dehydrogenase (LDH) showed no significant differences (all *p* > 0.05). Regarding lymphocyte subsets, no significant differences were found in the percentages of CD3^+^, CD3^+^CD4^+^, CD3^+^CD8^+^, CD3^−^CD19^+^, or CD3^−^CD(16^+^56^+^) cells between groups (all *p* > 0.05). Notably, the CD4^+^/CD8^+^ ratio was significantly lower in the cholestasis group (*p* < 0.05).

**Table 2:** Laboratory findings of infants with CMV infection.

Variable	Reference range	Cholestasis group (*n* = 64)	Non-cholestasis group (*n* = 64)	*p*
Hepatobiliary parameters				
TBil (μmol/L)	3.40–17.10	203.20 (176.87, 243.10)	110.85 (97.12, 130.22)	<0.001
IBil (μmol/L)	0–17.00	109.50 (85.70, 144.18)	86.95 (74.58, 109.95)	0.004
DBil (μmol/L)	0–6.80	72.90 (53.42, 94.58)	16.30 (13.40, 21.25)	<0.001
GGT (U/L)	9.00–150.00	171.50 (143.25, 198.25)	125.00 (67.75, 175.50)	<0.001
ALP (U/L)	98.00–532.00	316.00 (262.25, 429.25)	244.00 (178.75, 340.50)	<0.001
ALT (U/L)	8.00–71.00	32.66 (25.34, 41.44)	19.47 (13.44, 25.94)	<0.001
AST (U/L)	21.00–80.00	47.25 (39.80, 56.62)	38.05 (33.45, 45.85)	<0.001
Hematological parameters				
WBC (×10^9^/L)	4.30–14.20	9.66 ± 2.16	9.83 ± 2.33	0.668
Hemoglobin (g/L)	97.00–183.00	119.38 ± 12.81	122.64 ± 14.63	0.181
Platelets (×10^9^/L)	125.00–350.00	250.06 ± 69.13	234.22 ± 67.92	0.193
Eosinophils (%)	1.00–10.00	3.35 ± 1.36	3.76 ± 1.71	0.133
Neutrophils (×10^9^/L)	0.60–7.50	2.39 (1.39, 3.57)	2.21 (1.33, 3.03)	0.500
Lymphocytes (×10^9^/L)	2.40–9.50	5.94 ± 1.47	5.34 ± 1.66	0.031
Monocytes (×10^9^/L)	0.15–1.56	1.08 ± 0.31	0.99 ± 0.27	0.087
Other biochemical parameters				
Total protein (g/L)	49.00–71.00	49.94 ± 4.84	51.64 ± 4.48	0.041
Albumin (g/L)	35.00–50.00	32.00 ± 2.44	31.69 ± 2.54	0.480
CK (U/L)	25.00–200.00	214.80 (157.43, 273.22)	206.75 (144.62, 252.43)	0.234
Lipase (U/L)	0–60.00	12.10 (8.40, 16.43)	11.50 (8.47, 14.43)	0.487
CHE (U/L)	4000.00–13000.00	5206.56 ± 785.40	5472.34 ± 673.05	0.042
LDH (U/L)	120.00–246.00	626.88 ± 126.56	611.59 ± 102.74	0.454
Lymphocyte subsets				
CD3^+^	0.55–0.80	0.80 ± 0.06	0.79 ± 0.06	0.324
CD3^+^CD4^+^	0.30–0.60	0.59 (0.54, 0.63)	0.59 (0.53, 0.61)	0.715
CD3^+^CD8^+^	0.15–0.35	0.23 ± 0.06	0.22 ± 0.06	0.295
CD4^+^/CD8^+^	1.0–3.5	2.40 (1.80, 3.00)	2.90 (2.30, 3.60)	0.016
CD3^−^CD19^+^	0.08–0.30	0.08 (0.05, 0.12)	0.08 (0.05, 0.12)	0.617
CD3^−^CD (16^+^56)^+^	0.02–0.15	0.09 (0.06, 0.15)	0.09 (0.06, 0.12)	0.717

CMV, cytomegalovirus; TBil, total bilirubin; IBil, indirect bilirubin; DBil, direct bilirubin; GGT, gamma-glutamyl transferase; ALP, alkaline phosphatase; ALT, alanine aminotransferase; AST, aspartate aminotransferase; WBC, white blood cell count; CK, creatine kinase; CHE, cholinesterase; LDH, lactate dehydrogenase; NK, natural killer. Non-normally distributed continuous variables are expressed as median (interquartile range, IQR) and were compared using the Mann–Whitney U test; normally distributed continuous variables are expressed as mean ± SD and compared using the independent-sample t-test.

### 3.3 Comorbidities and complications in infants with CMV infection

Table 3 summarizes the clinical manifestations and complications of the infants with CMV infection. Overall, jaundice was the most common presenting manifestation, occurring in 103 of 128 infants (80.5%), followed by intracranial hemorrhage (44/128, 34.4%), neonatal anemia (34/128, 26.6%), neutropenia (29/128, 22.7%), and thrombocytopenia (28/128, 21.9%). Other manifestations included fever (19/128, 14.8%), poor weight gain (16/128, 12.5%), feeding difficulty (12/128, 9.4%), splenomegaly (8/128, 6.3%), and rash (6/128, 4.7%).

**Table 3:** Clinical manifestations and complications in infants with cytomegalovirus infection.

Variable	Cholestasis group (*n* = 64)	Non-cholestasis group (*n* = 64)	*p*
Jaundice, *n* (%)	64 (100.0)	39 (60.9)	<0.001
Jaundice onset (days of life)	3.00 (2.00, 7.25)	0.00 (0.00, 3.00)	<0.001
Duration of jaundice (days)	21.00 (11.00, 37.50)	0.00 (0.00, 9.00)	<0.001
Fever, *n* (%)	10 (15.6)	9 (14.1)	0.804
Feeding difficulty, *n* (%)	7 (10.9)	5 (7.8)	0.544
Rash, *n* (%)	3 (4.7)	3 (4.7)	1.000
Poor weight gain, *n* (%)	9 (14.1)	7 (10.9)	0.593
Neonatal anemia, *n* (%)	18 (28.1)	16 (25.0)	0.689
Passed hearing screening, *n* (%)	47 (73.4)	56 (87.5)	0.045
Normal fundus examination findings, *n* (%)	41 (64.1)	47 (73.4)	0.253
Splenomegaly, *n* (%)	5 (7.8)	3 (4.7)	0.492
Abnormal liver ultrasound, *n* (%)	17 (26.6)	1 (1.6)	<0.001
Intracranial hemorrhage, *n* (%)	26 (40.6)	18 (28.1)	0.137
Neutropenia, *n* (%)	15 (23.4)	14 (21.9)	0.833
Thrombocytopenia, *n* (%)	16 (25.0)	12 (18.8)	0.392
Length of hospital stay (days)	21.00 (13.00, 31.00)	12.50 (7.00, 20.25)	<0.001

Non-normally distributed continuous variables are expressed as median (interquartile range, IQR) and were compared using the Mann–Whitney U test; categorical variables are expressed as *n* (%) and compared using the *χ*^2^ test or Fisher’s exact test, as appropriate.

Compared with the non-cholestasis group, jaundice was significantly more frequent in the cholestasis group (100.0% vs. 60.9%, *p* < 0.05). Furthermore, infants in the cholestasis group had a later onset of jaundice and a longer duration of jaundice than those in the non-cholestasis group (all *p* < 0.05). No significant differences were observed between the two groups in the incidence of fever, feeding difficulty, rash, poor weight gain, neonatal anemia, splenomegaly, intracranial hemorrhage, neutropenia, thrombocytopenia, or normal fundus examination findings (all *p* > 0.05). However, the rate of passing the hearing screening was significantly lower in the cholestasis group than in the non-cholestasis group (73.4% vs. 87.5%, *p* < 0.05). In addition, abnormal liver ultrasound findings were more common in the cholestasis group (26.6% vs. 1.6%, *p* < 0.05). Infants with cholestasis also had a significantly longer length of hospital stay compared with those without cholestasis (21.0 [13.0–31.0] vs. 12.5 [7.0–20.3] days, *p* < 0.05).

### 3.4 Risk factors for cholestasis in infants with CMV infection and nomogram construction

To explore factors associated with cholestasis in infants with CMV infection, variables that differed significantly between groups and were clinically relevant were included in a multivariable logistic regression model, while hepatobiliary biochemical markers directly related to the diagnostic criteria for cholestasis were excluded to avoid circular reasoning. Gestational age was included as a forced covariate (Table 4). The results showed that breastfeeding was independently protective against cholestasis (adjusted OR [aOR]: 0.408, 95%CI: 0.159–0.840, *p* = 0.015), whereas delayed onset of jaundice was independently associated with cholestasis (aOR: 1.247, 95% CI: 1.101–1.412, *p* < 0.001). Additionally, lower total protein levels (aOR: 0.882, 95% CI: 0.797–0.976, *p* = 0.015) and decreased CD4^+^/CD8^+^ ratios (aOR: 0.589, 95% CI: 0.365–0.951, *p* = 0.030) were independently associated with cholestasis. Absolute lymphocyte count and cholinesterase were not statistically significant after adjustment.

**Table 4:** Factors associated with cholestasis in infants with CMV infection.

Variable	Crude OR (95% CI)	*p*	Adjusted OR (95% CI)	*p*
Gestational age (per week)	1.112 (0.944, 1.310)	0.205	1.119 (0.999, 1.253)	0.052
Breastfeeding	0.417 (0.175, 0.727)	0.005	0.408 (0.159, 0.840)	0.015
Jaundice onset (per day)	1.255 (1.117, 1.410)	<0.001	1.247 (1.101, 1.412)	<0.001
Lymphocyte count (×10^9^/L)	1.284 (1.020, 1.616)	0.034	1.224 (0.920, 1.630)	0.165
Total protein (g/L)	0.924 (0.855, 0.998)	0.044	0.882 (0.797, 0.976)	0.015
Cholinesterase (per 1,000 U/L)	0.605 (0.371, 0.988)	0.044	0.715 (0.387, 1.322)	0.285
CD4^+^/CD8^+^	0.651 (0.440, 0.963)	0.032	0.589 (0.365, 0.951)	0.030

CMV, cytomegalovirus; OR, odds ratio; CI, confidence interval. Adjusted ORs were obtained from multivariable logistic regression analysis. Gestational age was included as a forced covariate. Continuous variables were entered into the model according to clinically interpretable units. Variables with *p* < 0.05 in univariable analysis and clinical relevance were included in the multivariable model.

**Figure 1:**
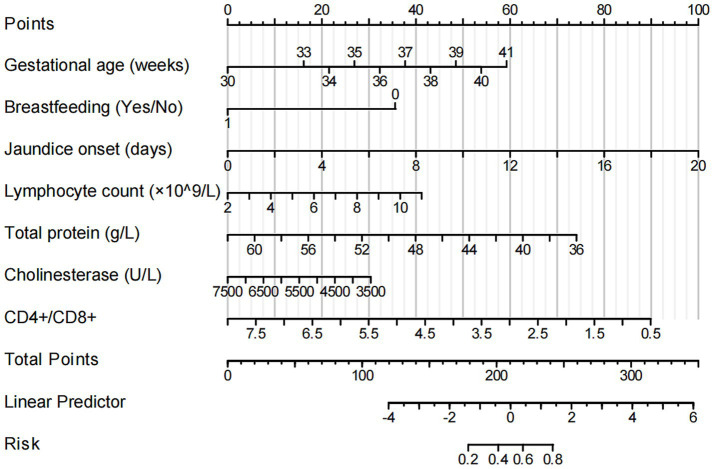
Nomogram for predicting the risk of cholestasis in infants with CMV infection. The nomogram was constructed based on the multivariable logistic regression model. Each variable is assigned a point value according to its regression coefficient: gestational age (weeks), breastfeeding (yes/no), jaundice onset (days), absolute lymphocyte count (×10^9^/L), total protein (g/L), cholinesterase (U/L), and CD4^+^/CD8^+^ ratio. The sum of points corresponds to the linear predictor, which is used to estimate the predicted probability of cholestasis. Higher total points indicate a greater risk of cholestasis. CMV, cytomegalovirus.

Based on the multivariable logistic regression model, a nomogram was constructed to estimate the probability of cholestasis in CMV-infected infants (Figure 1). Variables included in the nomogram were consistent with those in the regression model, and each variable was assigned a score proportional to its regression coefficient. The total score corresponds to the predicted probability of cholestasis for an individual infant. The model was statistically significant overall (*χ*^2^ = 46.628, *p* < 0.001) and demonstrated good discriminative ability with a concordance index (C-index) of 0.828 (95% CI, 0.757–0.899). Calibration assessed by the Hosmer–Lemeshow test showed no significant difference (*χ*^2^ = 5.234, *p* = 0.732), indicating good model calibration.

### 3.5 Phenotype axis analysis in infants with CMV infection

**Figure 2:**
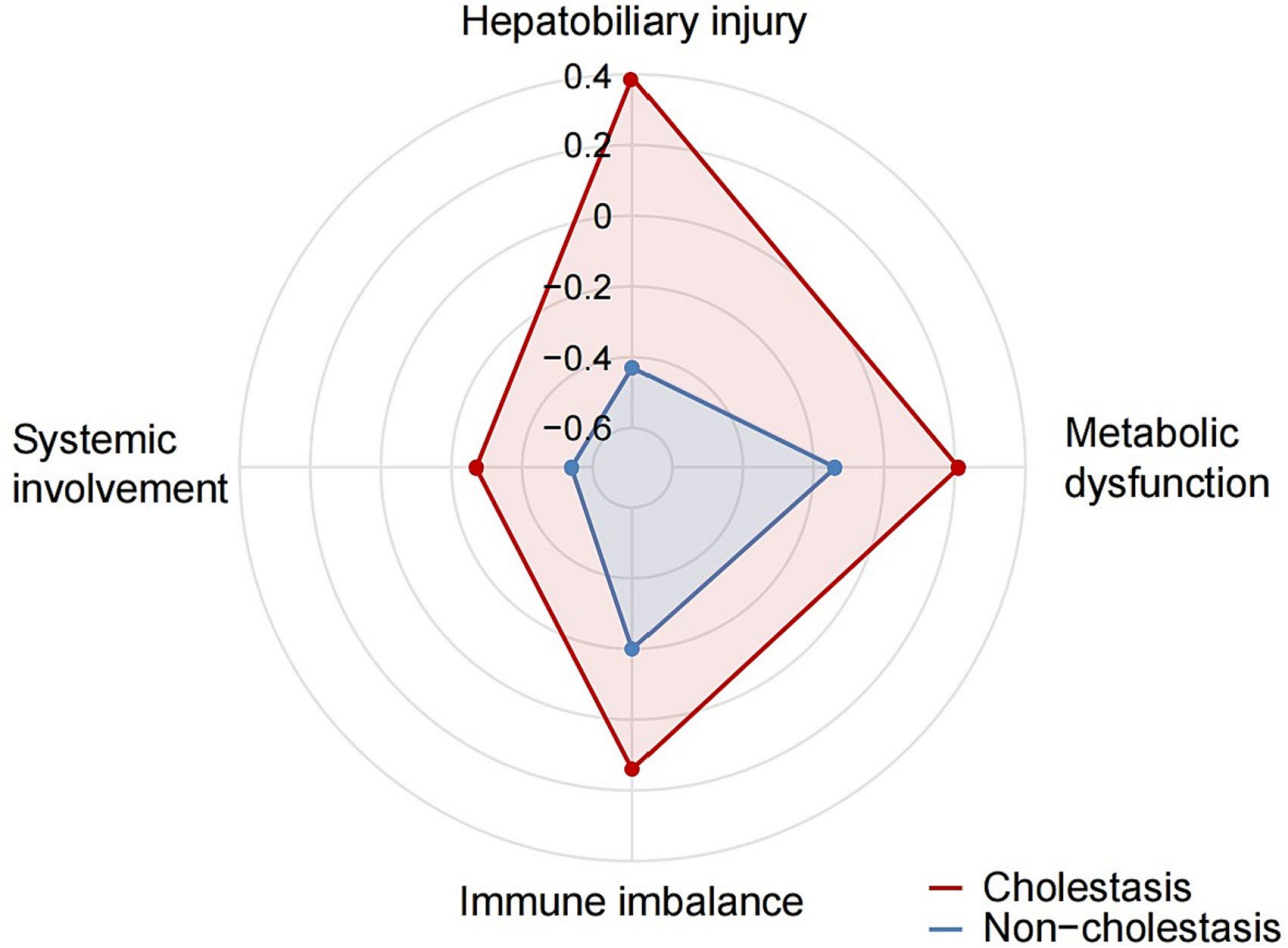
Radar plot of phenotype axis scores in infants with CMV infection. The radar plot depicts the median scores of four phenotype axes, hepatobiliary injury, metabolic dysfunction, immune imbalance, and systemic involvement, for infants with and without cholestasis. All variables were standardized using Z-scores, with higher values indicating a greater phenotypic burden. The red line (with red shaded area) represents infants in the cholestasis (case) group, and the blue line (with blue shaded area) represents infants in the non-cholestasis (control) group. Each axis on the radar plot corresponds to one of the four phenotype dimensions, and the larger the enclosed area, the greater the overall phenotypic burden. CMV, cytomegalovirus.

The phenotype axis analysis of infants with CMV infection is summarized in Table 5 and Figure 2. Infants with CMV infection and cholestasis exhibited higher phenotypic burden across multiple clinical dimensions. Compared with the non-cholestasis group, the cholestasis group showed the most pronounced differences in the hepatobiliary axis, followed by the metabolic axis, immune axis, and extrahepatic axis (all *p* < 0.05). Overall, the total phenotype burden was significantly higher in the cholestasis group than in the non-cholestasis group (*p* < 0.05).

**Table 5:** Comparison of phenotype axis scores between groups.

Phenotype axis	Cholestasis group	Non-cholestasis group	*p*
Hepatobiliary axis	0.39 (0.12–0.67)	−0.43 (−0.61 to 0.30)	<0.001
Metabolic axis	0.21 (−0.21–0.48)	−0.14 (−0.48 to 0.12)	<0.001
Immune axis	0.14 (−0.13–0.32)	−0.20 (−0.58 to 0.26)	0.007
Extrahepatic axis	−0.27 (−0.54–1.06)	−0.54 (−0.54 to 0.53)	0.031
Overall phenotype burden	0.26 (−0.04–0.46)	−0.23 (−0.48 to 0.00)	<0.001

Values are presented as median (interquartile range). Phenotype axis scores were calculated based on standardized variables (Z-scores) and are used for exploratory analysis. Higher scores indicate greater phenotypic burden. Phenotype axes include hepatobiliary axis, metabolic axis, immune axis, and extrahepatic axis.

## 4 Discussion

This study focused on young infants with CMV infection, a clinically heterogeneous population. Our results suggest that cholestasis should not be regarded solely as a biochemical manifestation of hepatobiliary dysfunction. Rather, it may reflect a broader disease phenotype characterized by hepatobiliary injury, disturbed bile acid metabolism, and immune perturbations. CMV possesses broad tissue tropism and can trigger organ-specific immune responses. Emerging evidence suggests that the extent and persistence of tissue damage are influenced not only by viral presence but also by the balance between host immune control and viral immune evasion mechanisms (Mihalić et al., 2024; Li et al., 2024). From this perspective, cholestasis may represent a clinically relevant marker of dysregulated host–virus interactions in CMV-infected infants, rather than merely an incidental finding accompanying viral detection.

In this study, delayed onset of jaundice was independently associated with cholestasis and was accompanied by prolonged duration of jaundice. Unlike transient physiological jaundice in the early postnatal period, the persistence and delayed onset of jaundice suggest a shift in the underlying pathology from simple immaturity of bilirubin metabolism to a cholestatic process primarily driven by impaired bile secretion and excretion (Feldman and Sokol, 2013). Mechanistically, infection-related cholestasis may be influenced by the release of inflammatory mediators, downregulation of canalicular transport proteins, disruption of bile acid homeostasis, and injury to the biliary epithelium; persistent bile acid retention can further exacerbate hepatocellular and biliary injury (Kriegermeier and Green, 2020; Karpen, 2020). Accordingly, delayed onset of jaundice in our cohort likely reflects progression from an early infection phase to a cholestasis-dominant hepatobiliary injury phase, rather than merely representing a temporal difference in presentation.

Lower total protein levels were independently associated with cholestasis, raising the possibility that impaired nutritional status and reduced hepatic synthetic function contribute to its pathogenesis. Interestingly, albumin concentrations were comparable between the two groups, suggesting that total protein may capture broader alterations in protein metabolism beyond albumin alone. As a composite indicator, total protein may more comprehensively reflect hepatic synthetic capacity, nutritional reserve, and systemic catabolic burden. In early infancy, when hepatic functional reserve is inherently limited, cholestasis may further impair the absorption of fats and fat-soluble nutrients, thereby establishing a vicious cycle in which nutritional deficits and hepatobiliary injury mutually reinforce one another (Heinz and Vittorio, 2023; Cimadamore et al., 2025). Another important finding of this study was that a decreased CD4^+^/CD8^+^ ratio was independently associated with cholestasis. The clinical manifestations of CMV infection largely depend on the host cellular immune status, particularly T-cell-mediated responses. Thus, a reduced CD4^+^/CD8^+^ ratio may reflect an alteration in T-cell response balance, suggesting a degree of immune dysregulation in infants with cholestasis (Mihalić et al., 2024; Shang and Li, 2024). Consistently, the exploratory phenotype axis analysis demonstrated a higher burden in the immune dysregulation dimension among infants with cholestasis, supporting the alignment of these findings.

Breastfeeding was identified as a protective factor in this study; however, these results should be interpreted with caution. Although breast milk may serve as a potential route for CMV transmission, current consensus does not negate its overall benefits, particularly in term and generally low-risk infants, and its role in immune modulation and early metabolic homeostasis is well recognized (Rawlinson et al., 2017; Lokossou et al., 2022). Therefore, our findings are more likely to indicate that breastfeeding in the context of CMV infection is associated with a more stable early immune and metabolic state, rather than representing a causal protective effect (Johnson et al., 2024). Given that this study did not further evaluate the duration of breastfeeding or milk-specific factors, these findings warrant confirmation in prospective studies.

Beyond conventional analyses based on individual clinical indicators, the phenotype axis framework revealed a substantially greater multidimensional disease burden among infants with cholestasis, encompassing hepatobiliary injury, metabolic disturbances, immune perturbations, and extrahepatic manifestations. This pattern suggests that CMV-associated cholestasis should be viewed as a systemic and multidimensional disease phenotype, rather than merely a biochemical manifestation of liver involvement. This concept aligns with recent research emphasizing the utility of multidimensional phenotyping to characterize complex disease features (Robinson, 2012), and is consistent with previous observations on CMV-related tissue injury and the role of immune responses in the pathogenesis of cholestasis (Mihalić et al., 2024; Wang et al., 2019; Jin et al., 2023). Nevertheless, this analysis remains exploratory, as its construction depended on predefined variables and grouping strategies, and it has not been externally validated. Therefore, the stability and applicability of these findings should be further evaluated in independent cohorts.

This study has several limitations. First, the single-center, retrospective case–control design may introduce selection and information biases. Second, the analysis was primarily based on routine clinical and laboratory parameters, without incorporating dynamic viral load measurements, bile acid profiles, or more detailed immunological indicators, limiting mechanistic interpretations. Third, because paired neonatal CMV IgM/IgG testing within the first 3 weeks of life or amniotic fluid PCR were not routinely performed, congenital and postnatally acquired CMV infection could not be reliably distinguished; this may have introduced some heterogeneity into the cohort, although clinical features suggested that the majority of cases represented postnatally acquired infection. In addition, the phenotype axis analysis was exploratory in nature; its construction relied on predefined variable groupings and equal weighting, introducing some subjectivity, and it lacked external validation and longitudinal data, so its stability and reproducibility remain uncertain. Therefore, the findings should be interpreted with caution and are intended primarily to highlight multidimensional clinical characteristics rather than to establish causal relationships.

## 5 Conclusion

CMV-infected infants with cholestasis displayed a distinct phenotype marked by prolonged jaundice, impaired hepatic synthetic and nutritional function, and immune alterations. Delayed jaundice onset, lower total protein levels, and reduced CD4+/CD8+ ratios were independently associated with cholestasis, whereas breastfeeding appeared to be protective. Multidimensional phenotype analysis further demonstrated a substantially greater phenotypic burden in infants with cholestasis. These findings provide new insights into the clinical spectrum of CMV-associated cholestasis and may aid in the early identification and management of high-risk infants.

## Data Availability

The original contributions presented in the study are included in the article/supplementary material, further inquiries can be directed to the corresponding authors.
